# Supplementary material for: Decoupling both local and global abundance from global range size, challenging the abundance-occupancy relationship in birds
Source: eLife. 2025 May 28;13:RP95857. doi: 10.7554/eLife.95857 (PMC12119083; doi:10.7554/eLife.95857)
Supplement: Supplementary file 1. [file elife-95857-supp1.docx]

**Supplementary file 1**

**Results of the intercept meta-analytic model using the *asreml* function**

| Effect | Name | Effect | SE | z value |
| --- | --- | --- | --- | --- |
|  |  |  |  |  |
| Fixed | Intercept | 0.0145081 | 5.17e-03 | 2.80459 |
| Random | Country | -0.528 | 5.76e-04 | 7.915738 |
| Random | State code | 0.005161983 | 1.96e-04 | 26.331084 |
| Random | Effect size (units) | 0.001597069 | 2.22e-05 | 71.864656 |
|  |  |  |  |  |

**Results of the meta-regression model with ‘checklist duration’ as a moderator, using the *asreml* function**

| Effect | Name | Effect | SE | z value |
| --- | --- | --- | --- | --- |
|  |  |  |  |  |
| Fixed | Intercept | 0.02314897 | 5.16E-03 | 4.490484 |
| Fixed | z(ln(checklist duration)) | -0.019646 | 7.20E-05 | -272.88526 |
| Random | Country | 0.00451859 | 5.72E-04 | 7.894702 |
| Random | State code | 0.00519698 | 1.97E-04 | 26.372854 |
| Random | Effect size (units) | 0.00127717 | 2.20E-05 | 57.961243 |
|  |  |  |  |  |

**Results of the meta-regression model with ‘sampling variance’ as a moderator, using the *asreml* function**

| Effect | Name | Effect | SE | z value |
| --- | --- | --- | --- | --- |
|  |  |  |  |  |
| Fixed | Intercept | -0.0171537 | 5.23E-03 | -3.279982 |
| Fixed | Sampling variance | 0.36149029 | 1.47E-03 | 245.747022 |
| Random | Country | 0.00466502 | 5.89E-04 | 7.91356 |
| Random | State code | 0.00530528 | 2.01E-04 | 26.4252 |
| Random | Effect size (units) | 0.00143218 | 2.21E-05 | 64.76545 |
|  |  |  |  |  |

**Results of the meta-regression model with ‘checklist duration’ and ‘sampling variance’ as moderators, using the *asreml* function**

| Effect | Name | Effect | SE | z value |
| --- | --- | --- | --- | --- |
|  |  |  |  |  |
| Fixed | Intercept | 0.00089738 | 5.19E-03 | 0.1728713 |
| Fixed | z(ln(checklist duration)) | -0.0147034 | 8.01E-05 | -183.44948 |
| Fixed | Sampling variance | 0.22952897 | 1.64E-03 | 140.287413 |
| Random | Country | 0.004581054 | 5.80E-04 | 7.896078 |
| Random | State code | 0.005273559 | 2.00E-04 | 26.418513 |
| Random | Effect size (units) | 0.001272852 | 2.20E-05 | 57.801132 |
|  |  |  |  |  |
