# Supplementary material for: Decoupling both local and global abundance from global range size, challenging the abundance-occupancy relationship in birds
Source: eLife. 2025 May 28;13:RP95857. doi: 10.7554/eLife.95857 (PMC12119083; doi:10.7554/eLife.95857)
Supplement: Supplementary file 2. [file elife-95857-supp2.docx]

**Supplementary file 2**

**Results of the phylogenetic regression using the *phylolm* and *miInference* functions**

| Effect | Name | Effect | SE | t value (df) |
| --- | --- | --- | --- | --- |
|  |  |  |  |  |
| Fixed | Intercept | -0.91553 | 1.6476 | -0.556 (1776.3) |
| Fixed | log10(range size) | 0.022645 | 0.092832 | 0.244 (99.6) |
|  |  |  |  |  |
