# Supplementary material for: Decoupling both local and global abundance from global range size, challenging the abundance-occupancy relationship in birds
Source: eLife. 2025 May 28;13:RP95857. doi: 10.7554/eLife.95857 (PMC12119083; doi:10.7554/eLife.95857)
Supplement: Supplementary file 3. [file elife-95857-supp3.docx]

**Supplementary file 3**

**Notations of statistical models used in this study (results of these models are in Supplementary file 1)**

| Fromula | Variance components (normally distributed with the mean of 0 and given $\sigma^{2}$ values |
| --- | --- |
| $Zr_{i}=\beta_{0}+\text{County}_{k\left[ i \right]}+\text{State-code}_{j\left[ i \right]}+\text{Effect-size}_{i}$ | $\text{County}_{k}\mathcal{\sim N}\left( 0,\sigma_{country}^{2} \right)$  $\text{State-code}_{j}\mathcal{\sim N}\left( 0,\sigma_{State-code}^{2} \right)$  $\text{Effect-size}_{i}\mathcal{\sim N}\left( 0,\sigma_{Effect-size}^{2} \right)$  $\text{Sampling-error}_{i}\mathcal{\sim N}\left( 0,\sigma_{Sampling-error_{i}}^{2} \right)$ |
| $Zr_{i}=\beta_{0}+\beta_{1}*\text{z(ln(checklist duration))}_{i}+\text{County}_{k\left[ i \right]}+\text{State-code}_{j\left[ i \right]}+\text{Effect-size}_{i}$ | $\text{County}_{k}\mathcal{\sim N}\left( 0,\sigma_{country}^{2} \right)$  $\text{State-code}_{j}\mathcal{\sim N}\left( 0,\sigma_{State-code}^{2} \right)$  $\text{Effect-size}_{i}\mathcal{\sim N}\left( 0,\sigma_{Effect-size}^{2} \right)$  $\text{Sampling-error}_{i}\mathcal{\sim N}\left( 0,\sigma_{Sampling-error_{i}}^{2} \right)$ |
| $Zr_{i}=\beta_{0}+\beta_{1}*\text{Sampling-variance}_{i}+\text{County}_{k\left[ i \right]}+\text{State-code}_{j\left[ i \right]}+\text{Effect-size}_{i}$ | $\text{County}_{k}\mathcal{\sim N}\left( 0,\sigma_{country}^{2} \right)$  $\text{State-code}_{j}\mathcal{\sim N}\left( 0,\sigma_{State-code}^{2} \right)$  $\text{Effect-size}_{i}\mathcal{\sim N}\left( 0,\sigma_{Effect-size}^{2} \right)$  $\text{Sampling-error}_{i}\mathcal{\sim N}\left( 0,\sigma_{Sampling-error_{i}}^{2} \right)$ |
| $Zr_{i}=\beta_{0}+\beta_{1}*\text{z(ln(checklist duration))}_{i}+\beta_{2}*\text{Sampling-variance}_{i}+\text{County}_{k\left[ i \right]}+\text{State-code}_{j\left[ i \right]}+\text{Effect-size}_{i}$ | $\text{County}_{k}\mathcal{\sim N}\left( 0,\sigma_{country}^{2} \right)$  $\text{State-code}_{j}\mathcal{\sim N}\left( 0,\sigma_{State-code}^{2} \right)$  $\text{Effect-size}_{i}\mathcal{\sim N}\left( 0,\sigma_{Effect-size}^{2} \right)$  $\text{Sampling-error}_{i}\mathcal{\sim N}\left( 0,\sigma_{Sampling-error_{i}}^{2} \right)$ |
| $\text{Abundance}_{i}=\beta_{0}+\beta_{1}*\text{log10(range size)}+\text{Error}_{i}$ | $\text{Error}_{i}\mathcal{\sim N}\left( 0,\sigma_{error}^{2}\mathbf{A} \right)$ |
|  |  |

$Zr_{i}$ denotes the *i*th effect size, $\beta_{0}$ is the intercept, $\beta_{1}$ and $\beta_{2}$ are regression coeffects, $\text{County}_{k}$ is the kth country, $\text{State-code}_{j}$ is the *j*th state code and $\text{Effect-size}_{i}$ is the *i*th effect size, and z(ln(checklist duration)), Sampling-variance and log10(range size) are fixed effects (predictors; see Method).
